# Vaccination as a social contract

**DOI:** 10.1073/pnas.1919666117

**Published:** 2020-06-15

**Authors:** Lars Korn, Robert Böhm, Nicolas W. Meier, Cornelia Betsch

**Affiliations:** ^a^Media and Communication Science, University of Erfurt, 99089 Erfurt, Germany;; ^b^Center for Empirical Research in Economics and Behavioral Sciences, University of Erfurt, 99089 Erfurt, Germany;; ^c^Department of Psychology, University of Copenhagen, 1353 Copenhagen, Denmark;; ^d^Department of Economics, University of Copenhagen, 1353 Copenhagen, Denmark;; ^e^Copenhagen Center for Social Data Science (SODAS), University of Copenhagen, 1353 Copenhagen, Denmark;; ^f^School of Business and Economics, RWTH Aachen University, 52062 Aachen, Germany

**Keywords:** vaccine decision making, generosity, reciprocity, vaccine advocacy, vaccine hesitancy

## Abstract

Most vaccines protect both the vaccinated individual and the society by reducing the transmission of infectious diseases. In order to eliminate infectious diseases, individuals need to consider social welfare beyond mere self-interest—regardless of ethnic, religious, or national group borders. It has therefore been proposed that vaccination poses a social contract in which individuals are morally obliged to get vaccinated. However, little is known about whether individuals indeed act upon this social contract. If so, vaccinated individuals should reciprocate by being more generous to a vaccinated other. On the contrary, if the other doesn’t vaccinate and violates the social contract, generosity should decline. Three preregistered experiments investigated how a person’s own vaccination behavior, others’ vaccination behavior, and others’ group membership influenced a person’s generosity toward respective others. The experiments consistently showed that especially compliant (i.e., vaccinated) individuals showed less generosity toward nonvaccinated individuals. This effect was independent of the others’ group membership, suggesting an unconditional moral principle. An internal metaanalysis (*n* = 1,032) confirmed the overall social contract effect. In a fourth experiment (*n* = 1,212), this pattern was especially pronounced among vaccinated individuals who perceived vaccination as a moral obligation. It is concluded that vaccination is a social contract in which cooperation is the morally right choice. Individuals act upon the social contract, and more so the stronger they perceive it as a moral obligation. Emphasizing the social contract could be a promising intervention to increase vaccine uptake, prevent free riding, and, eventually, support the elimination of infectious diseases.

## Significance

Vaccines support controlling and eliminating infectious diseases. As most vaccines protect both vaccinated individuals and the society, vaccination is a prosocial act. Its success relies on a large number of contributing individuals. We study whether vaccination is a social contract where individuals reciprocate and reward others who comply with the contract and punish those who don’t. Four preregistered experiments demonstrate that vaccinated individuals indeed show less generosity toward nonvaccinated individuals who violate the social contract. This effect is independent of whether the individuals are members of the same or different social groups. Thus, individuals’ behavior follows the rules of a social contract, which provides a valuable basis for future interventions aiming at increasing vaccine uptake by emphasizing this social contract.

Measles has reemerged with full force: in the first half of 2019, 364,808 measles cases were recorded in 182 countries—the highest number since 2006 (1). The 2019/2020 outbreak in Samoa caused over 80 deaths, mainly children (2). The insufficient uptake of the measles-containing vaccine is a major threat to individual and global health, such that the World Health Organization (WHO) termed vaccine hesitancy as one of the 10 major threats to public health in 2019 (3). As a consequence, mandatory vaccination policies have been discussed and introduced in several countries [e.g., Italy, France, Germany (4–6) see ref. 7 for an overview). In Germany, for example, specific population groups, such as preschool children, migrants, and asylum seekers, are required to prove that they have been vaccinated against measles since 1 March 2020.

Mandates often elicit emotional public debates that weigh freedom of choice against social welfare concerns. The German Ethics Committee has made a strong case against mandates (8). At the same time, the committee has stressed that getting vaccinated is a moral obligation in the sense that vaccination constitutes a social contract that every individual is morally obliged to obey (8). This stance is justified due to the social benefit of vaccines. As most vaccines also reduce the transmission of a disease, they indirectly protect the community and individuals who are too young to get vaccinated or immunocompromised (9) (“herd immunity” or “community immunity”). Hence, the social contract results from the moral obligation to protect vulnerable others.

However, the interplay of such indirect effects of vaccination and the costs associated with vaccination (e.g., time, effort, risk of vaccine-adverse events) constitutes a social dilemma, in which collective and individual interests are at odds (refs. 10 and 11; for an interactive simulation, see ref. 12). Therefore, individuals have incentives to refrain from vaccination and to free ride by profiting from others’ indirect protection, thus, to selfishly breaking the social contract.

In this study, we therefore investigate whether individuals’ behavior suggests that vaccination is indeed perceived as a social contract. The hypotheses are directly derived from the social contract perspective, which we put to a critical empirical test.

## Theoretical Background and Hypotheses.

**Fig. 1.**
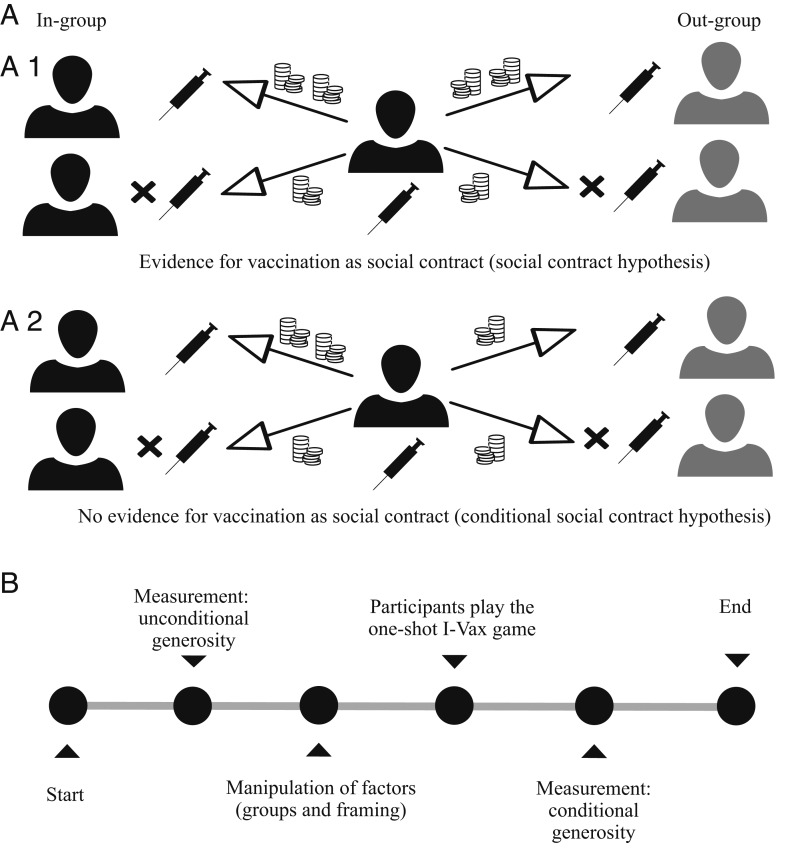
Illustration of the experimental setting (*A*) and procedure (*B*) used to test whether vaccination is a social contract. (*A*) After learning whether the other person vaccinated or did not vaccinate in the experimental game, the participants (*Center*) allocated money between oneself and four other people, respectively, who were either vaccinated or not and belonged to the in-group (black) or an out-group (gray). Changes to a baseline measure indicated changes in generosity. *A.1* describes the situation in which vaccination is a social contract: vaccinated individuals allocate more money to vaccinated others and less money to nonvaccinated others (social contract hypothesis). In *A.1*, changes in generosity apply to all others alike, i.e., changes are independent from the others’ group memberships. *A.2*, however, describes a case where the decision maker changes his or her generosity only with regard to in-group but not to out-group members (conditional social contract). This pattern would indicate that vaccination is not a social contract.

The morality-as-cooperation theory (13) postulates that morality is a container of behaviors to solve cooperation problems. More specifically, cooperation is considered as morally good and respected, whereas defection is seen as morally bad and despised. This perception should be especially pronounced among individuals who are cooperators themselves (14). This means that cooperators in particular have reciprocity concerns: “I’ll scratch your back, you’ll scratch mine” (15). Thus, given others cooperate, cooperators should share their resources generously. However, when this reciprocity expectation is violated, cooperators should become less generous. If we transfer this to the vaccination context, individuals who are vaccinated (and therefore comply with the social contract) should show more generosity toward those who are vaccinated compared to those who are not vaccinated (and therefore violate the social contract). Importantly, individuals who are not vaccinated themselves should not (or to a smaller extent) differentiate between the other persons’ vaccination behaviors. We refer to this as the social contract hypothesis. Fig. 1*A.1* illustrates this for a vaccinated individual.

Moral norms are considered universal principles of human interaction (13, 16). Thus, the rules inherent in a social contract should apply to all individuals irrespective of their group membership (Fig. 1*A.1*). For example, (non)vaccinated migrants from another country (i.e., out-group members) and (non)vaccinated members from the same country (i.e., in-group members) should induce equal levels of generosity. However, previous research showed that individuals treat in-group members more positively than out-group members (intergroup bias, refs. 17–19). Therefore, we tested whether the social contract applies only to in-group members or whether it is moderated by group membership (19). We refer to this as the conditional social contract hypothesis (Fig. 1*A.2*). The hypothesis thus challenges the idea of vaccination as a social contract as it contrasts the idea of moral norms—which should apply to all people alike—with group- and context-specific reactions toward others’ vaccination behavior. In addition to its theoretical importance, the question also has practical relevance, as societies are growing more and more diverse through global migration movements (20), which makes intergroup aspects especially relevant.

## Assessing Generosity toward (Non)Vaccinated Individuals.

An obvious solution to assess reciprocal behavior within a social contract setting would be to test whether people are more likely to vaccinate given that others vaccinate as well. Indeed, individuals condition their own vaccination decision on the (expected) vaccination decisions of others (11, 21–23). However, one may also decide not to be vaccinated and instead free ride when the protection by the “herd” is sufficient (11). The incentives for vaccination are low, for example, when the vaccine uptake is already high and there are some costs associated with vaccination. A growing body of evidence shows that vaccination behavior is affected by strategic considerations responding to the changes in incentives, given different levels of vaccine uptake (11, 24). Thus, due to these strategic considerations, the vaccination intention or behavior, given a certain level of uptake, will not be a meaningful indicator for reciprocity.

We therefore assessed generosity, i.e., tested whether individuals reciprocate and reward others who comply with the contract and punish those who do not do so, by using an established and incentivized measure of social preferences (25). In this task, participants allocate monetary tokens between themselves and others (Fig. 1) which indicates their baseline level of generosity, i.e., the money a “neutral” other can expect from the participant. We then compared whether the money allocated to the other increased or decreased relative to the baseline after participants played an interactive vaccination game and had learned about the vaccination decision and group membership of the other. An increase of money allocated to the other indicates higher generosity, less money allocated indicates lower generosity. Generosity in this measure is independent from the incentives in the vaccination decision task and thus yields a strong test of the social contract hypothesis.

## Overview of the Experiments and Internal Metaanalysis.

In the first three incentivized and preregistered online experiments, we collected data from *n* = 1,032 participants to test the social contract hypothesis. First, the participants indicated their unconditional generosity by distributing monetary tokens between themselves and an unknown other participant. Fig. 1*B* provides a general overview of the procedure. Afterward, they were informed that two groups existed during the experiment and that they were assigned to one of them. As framing may alter behavior (26), experiment 3 further varied the framing of the decision situation by using a migration context for the intergroup setting: instead of being a member of group A or B as in experiments 1 and 2, participants were informed that they were citizens of a country vs. immigrants from another country in experiment 3. The participants then made a vaccination decision in an incentivized vaccination game (one-shot I-Vax game; refs. 11 and 27), which models vaccinations based on epidemiologically derived incentives capturing both the direct and indirect benefits as well as the costs of vaccination. Each decision in the game has real monetary consequences: becoming sick means a loss of money, and the fewer people are vaccinated, the higher is one’s likelihood to get sick; however, vaccination also leads to small fixed costs and can result in an additional loss of money when side effects occur. After the vaccination game, the participants again indicated their generosity, but this time they received additional information about the other person’s vaccination behavior and group membership. Main dependent variable was the change in (reciprocal) generosity, i.e., how generous participants were to the respective others as compared to their baseline level of generosity. Hence, the experiments used a 2 (participant’s vaccination decision: nonvaccination vs. vaccination; quasiexperimental between-subjects) × 2 (other’s vaccination decision: nonvaccination vs. vaccination; within-subjects) × 2 (other’s group membership: in-group vs. out-group; within-subjects) design.

When vaccinated participants become more generous to vaccinated than to nonvaccinated others and unvaccinated participants do not make this distinction, we interpret this as evidence for the social contract hypothesis. Moreover, assessing whether such changes depend on the group membership of the other person challenges the idea of vaccination being a moral social contract, as moral rules should apply to all people alike. Experiments 2 and 3 also assessed how warm or cold one feels toward others (“perceived warmth”), as this fundamental evaluation of others corresponds to a moral judgment of others’ behavior (28); this allowed an additional test of the hypotheses. An internal metaanalysis tested the hypotheses across all three experiments.

A fourth incentivized and preregistered online experiment (*n* = 1,212) tested whether the pattern of results occurred due to the incentive structure of the decision task or the contextual framing as a vaccination decision. Participants hence decided in favor of vaccination and against vaccination in the vaccination-framing condition, and in favor of option A and against option A in the neutral framing condition. Moreover, some infectious diseases are noncommunicable and vaccination does not lead to herd immunity, such as in the case of tetanus (29). Therefore, the fourth experiment also tested whether vaccination is a social contract only when herd immunity plays a role and players’ outcomes are mutually dependent. The experiment thus implemented a 2 (participant’s decision: nonvaccination [not option A] vs. vaccination [option A]; quasiexperimental between-subjects) × 2 (other’s decision: nonvaccination [not option A] vs. vaccination [option A]; within-subjects) × 2 (framing: neutral vs. vaccination, between-subjects) × 2 (mutual dependence: absent vs. present, between-subjects) mixed design to investigate changes in generosity. We also explored whether the belief that vaccination is a moral obligation moderates the expected changes in generosity.

## Results

**Fig. 2.**
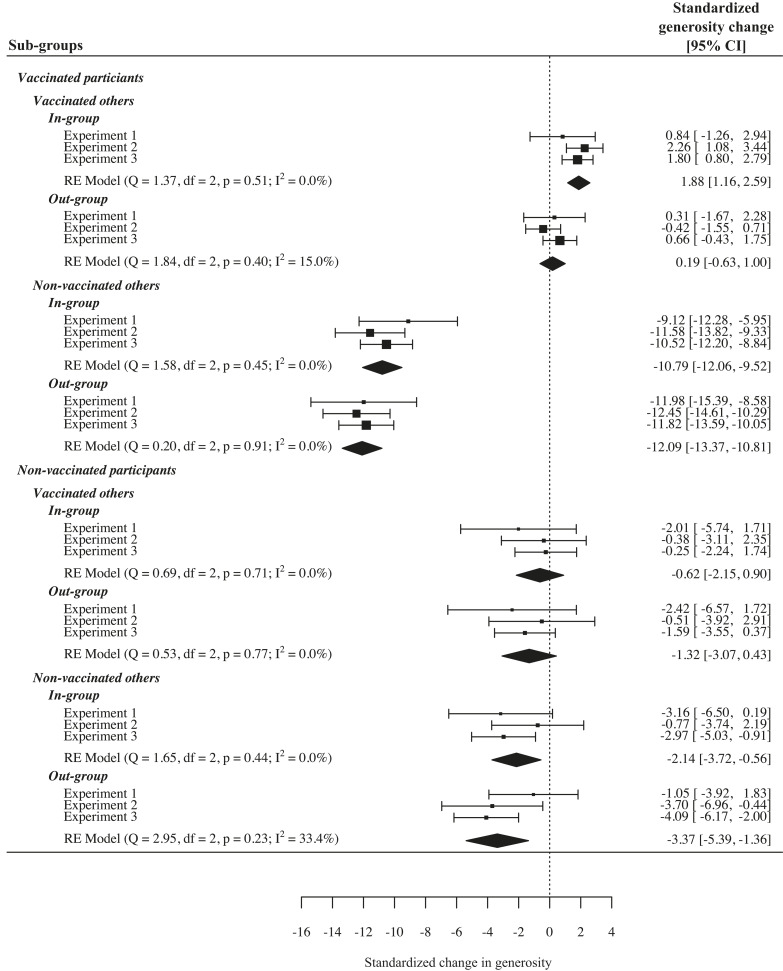
Forest plot of mean changes in generosity (generosity conditional to own and other decision and group membership, relative to the unconditional baseline). Positive values indicate an increase in generosity, whereas negative values indicate a decrease in generosity. Absolute values of unconditional baseline generosity can be found in *SI Appendix*, Table S8. The pattern of results shows that the interaction effect between participants’ vaccination decisions and others’ vaccination decisions (social contract hypothesis) was mainly driven by a reduction of generosity from vaccinated participants toward nonvaccinated others. Nonvaccinated participants also showed a reduction of generosity toward nonvaccinated others, but this effect was smaller than among vaccinated participants. An increase in generosity was less pronounced and was only shown from vaccinated participants toward vaccinated in-group members. Note: Changes in generosity refer to standardized changes. The experimental materials were comparable and varied only in little details as described in the methods section; thus, the three experiments can be understood as conceptual replications. The overall effects were calculated using a random effects model for meta-analysis. Q and I^2^ were used for heterogeneity assessment among the studies. CIs refer to 95% confidence intervals. *N*_Experiment__1_ = 117, *N*_Experiment__2_ = 372, *N*_Experiment__3_ = 444.

**Fig. 3.**
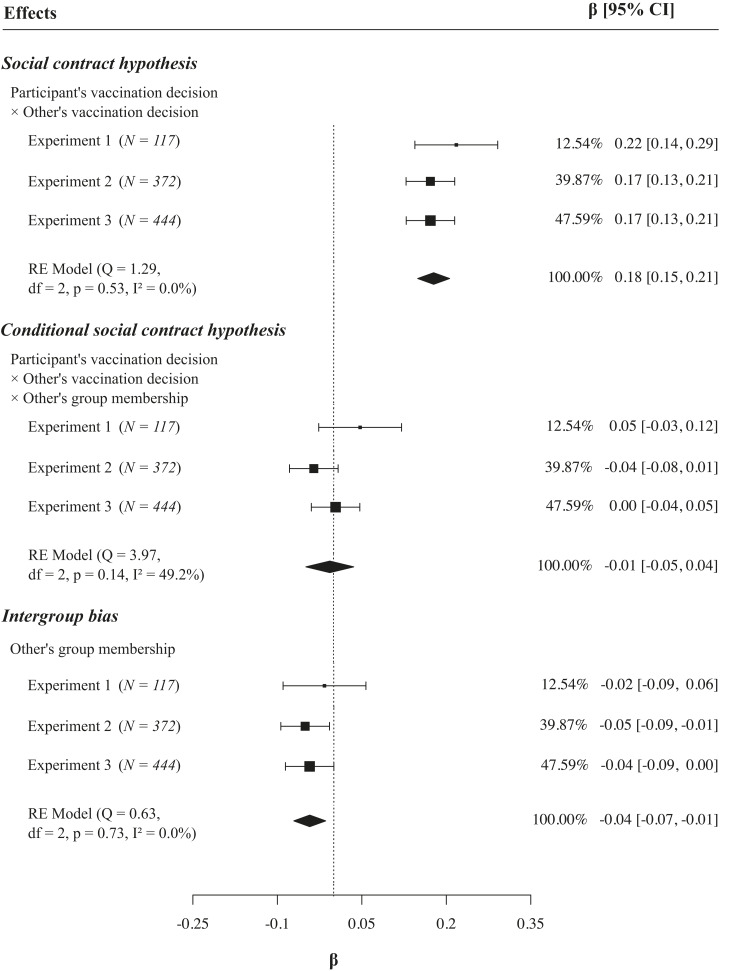
Forest plot displaying tests of the social contract and conditional social contract. Hypotheses across three experiments. The results show evidence for the social contract hypothesis as indicated by the significant interaction effect of the participant’s and the other’s vaccination decision (*Top*). There was no evidence for the conditional social contract hypothesis as indicated by the nonsignificant interaction effect (*Middle*) between participant’s and other’s decision and group membership – this supports the idea of vaccination being a social contract irrespective of the individuals’ group memberships. The manipulation check (*Bottom*) confirmed that the groups were indeed perceived as distinct groups by showing a significant main effect for group membership. *Note:* *SI Appendix*, Fig. S1 in the supplement displays all remaining main effects and interaction effects of the analysis. The experimental materials were comparable and varied only in little details as described in *Materials and Methods*; thus, the three experiments can be understood as conceptual replications. The figure display betas, calculated from mixed effects regressions, and overall effects using a random effects model for metaanalysis. Q and I^2^ were used for heterogeneity assessment.

Figs. 2 and 3 and *SI Appendix*, Fig. S1 show the results of the random effects metaanalysis. The results support the social contract hypothesis: as can be seen in the upper half of Fig. 2, across all three experiments, vaccinated participants were sensitive to others’ vaccination decisions and reduced their generosity toward nonvaccinated others as compared to vaccinated others. As displayed in the lower half of Fig. 2, nonvaccinated participants differentiated less between vaccinated and nonvaccinated others (overall interaction effect β = 0.18, *P* < 0.001; Fig. 3). As can further be seen in Fig. 2, the absolute decrease in generosity toward nonvaccinated others was larger than the absolute increase in generosity toward vaccinated others; the latter was only shown by vaccinated individuals. Thus, vaccinated individuals changed their generosity based on others’ vaccination behavior, while unvaccinated participants did not, providing evidence for vaccination as a social contract.

In order to test whether the social contract depends on group memberships, it first had to be confirmed that the groups were indeed perceived as distinct groups and thus the manipulation was successful. Indeed, supporting previous research, there was a significant intergroup bias (17–19), which was indicated by larger generosity toward in-group members than toward out-group members (β = −0.04, *P* = 0.003; Fig. 3).

Further, and more importantly, the analysis provided no evidence for the conditional social contract hypothesis: the reduction of generosity among vaccinated participants was not more pronounced toward nonvaccinated out-group members than toward nonvaccinated in-group members (β = −0.01, *P* = 0.761; Fig. 3). This independence from group memberships further supports the idea of vaccination being a social contract as the social contract applies to all people alike.

Experiments 2 and 3 additionally assessed the perceived warmth conditional on the others’ characteristics as measured after playing the I-Vax game but before assessing conditional generosity. *SI Appendix*, Table S2 shows the results of a mixed effects regression with participants’ vaccination decisions, others’ vaccination decision, and group membership as experimental factors. The regression also included the experiment as a factor to account for the variation in materials between the experiments. Overall, the analysis of perceived warmth replicated the above pattern of results: Especially vaccinated participants showed less warmth toward nonvaccinated others as compared to vaccinated others. Nonvaccinated participants again differentiated less between vaccinated and nonvaccinated others (interaction effect: β = 0.22, *P* < 0.001). Also, there was a significant intergroup bias: participants felt less warmth toward out-group members compared to in-group members (β = −0.08, *P* < 0.001). Again, whether vaccinated participants felt more or less warm toward vaccinated and nonvaccinated others was independent from the others’ group membership (β = −0.03, *P* = 0.078).

**Fig. 4.**
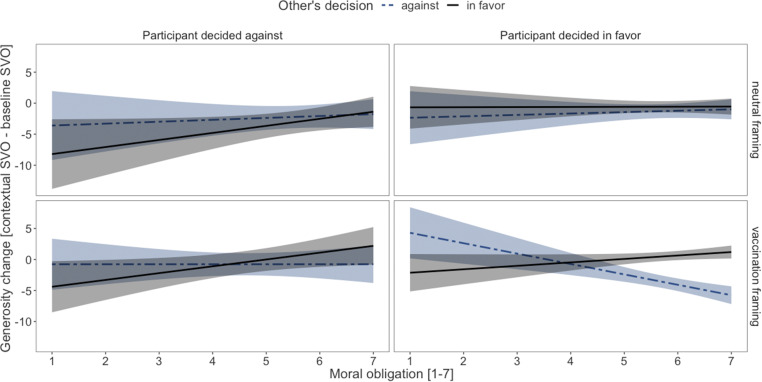
Testing the social contract hypothesis in a task framed as neutral or as vaccination decision and depending on the perception of vaccination as a social contract. The figure displays changes in generosity as a function of participant’s vaccination decision (columns), other’s vaccination decision (lines), framing (*Top* vs. *Bottom*), and perceiving vaccination as a moral obligation (*x* axis) in experiment 4. Conditional generosity is indicated by the black line being above the gray line (increased generosity toward vaccinated and decreased generosity toward unvaccinated individuals). When framed as a vaccination decision, vaccinated participants were less generous to nonvaccinated others the more they perceived vaccination as a social contract; likewise, generosity toward vaccinated others increased with increasing perception of vaccination as a social contract (*Lower Right* quadrant). Regardless of framing, nonvaccinated participants again did not differentiate between vaccinated and nonvaccinated others, irrespective of their belief of vaccination as social contract (*Lower Left* quadrant). This pattern again supports the idea of vaccination as a social contract. Note: Confidence bands represent 95% CI. *n* = 1,212.

Experiment 4 tested boundary conditions of the social contract hypothesis and investigated whether contextual framing, mutual dependence between individuals, and beliefs that vaccination is a social contract moderate the changes of generosity of vaccinated individuals toward vaccinated vs. nonvaccinated others. *SI Appendix*, Table S7 shows the results of a mixed effects regression with participants’ decisions, others’ decisions, framing of the decision task, mutual dependence, and perceptions of vaccination as a social contract as independent variables on changes in generosity. Experiment 4 again provides evidence for the social contract hypothesis: Participants who decided in favor of vaccination (or in favor of option A) reacted sensitively toward others’ decisions and reduced their generosity toward those who decided against vaccination (or against option A) but not to those who decided in favor of vaccination (or in favor of option A). Participants who decided against vaccination (or against option A) differentiated less between others based on their decision (interaction effect: β = 0.05, *P* = 0.019; *SI Appendix*, Fig. S5). This effect did not depend on the framing of the decision task or on mutual dependence (both three-way interactions, *ns*). Interestingly, however, the individual perception of how much vaccination was seen as a moral obligation played a role, but only when the task was framed as a vaccination decision (four-way interaction: own and other’s decision, framing, and beliefs of vaccination as a moral obligation, β = 0.04, *P* = 0.034; Fig. 4). The pattern yields additional evidence for the idea that vaccination is a social contract, as the conditional changes in generosity were especially pronounced, the stronger the participants perceived vaccination as a moral obligation (*Lower Right* quadrant): vaccinated participants showed less generosity toward nonvaccinated others the more they perceived vaccination as a moral obligation; likewise, generosity toward vaccinated others increased with increasing perception of vaccination as a moral obligation. Regardless of framing and irrespective of their belief of vaccination as a moral obligation, participants who decided against vaccination (or against option A) again did not differentiate between vaccinated and nonvaccinated others (and between those who decided in favor or against option A; *Left* quadrants).

## Discussion

The social dilemma of vaccination sometimes puts individual interests at odds with the societal goal of eliminating infectious diseases. The present research supports the notion that vaccination is a social contract wherein getting vaccinated is the morally right behavior. Four preregistered experiments investigated whether individuals’ changes in generosity suggest that vaccination is a social contract based on a moral obligation. There was consistent evidence that vaccinated participants (more so than nonvaccinated participants) showed lower levels of generosity toward nonvaccinated others, representing the behavioral foundation of a social contract. Furthermore, vaccinated individuals showed lower levels of generosity toward nonvaccinated others regardless of their group membership. We interpret this finding as an indicator of an unconditional moral principle. This is backed up by the finding that the effects also replicated for perceived warmth as a dependent variable, as warmth corresponds to a moral judgment of others’ behavior (28). The current results also relate to findings on the avoidance tendency of the behavioral immune system, where individuals tend to avoid others based on their actual infection status but not on mere group memberships (30). As the current outbreak of COVID-19 in China shows, however, this may only be true as long as the origin of the disease is not associated with group membership.

The fourth experiments explicitly examined whether perceiving vaccination as a moral obligation has an impact on the observed patterns. The results revealed that this belief is indeed a major driver among vaccinated individuals to show less generosity toward nonvaccinated others (vs. vaccinated others). The pattern also suggests that the effects are rather specific for vaccination as framing mattered, particularly for individuals who perceive vaccination as a moral obligation. As we did not find an effect of the mutual dependence (i.e., whether a vaccine leads to herd immunity), we conclude that the effect is robust across different incentives for (non)vaccination. As only a small number of vaccines do not provide herd immunity, this situation may be so rare that the vaccination framing triggers common knowledge about vaccination, which may include the information about herd immunity. Importantly, the results replicated especially in the situation closest to reality, which is encouraging for a potential application of the findings. Thus, we conclude that vaccination is a social contract in which cooperation is the morally right choice. Individuals act upon the social contract, and more so the stronger they perceive it as a moral obligation.

### Practical Implications.

Emphasizing that vaccination is a social contract seems to be a promising extension of communicating the social benefit of vaccination (22). Accordingly, stressing that everyone who is able to get vaccinated is expected to do so could have additional benefits in communicating the principle of herd immunity. The appeal could be based on moral grounds (e.g., stating that violating the social contract has a negative impact on vulnerable demographic groups and thus on the health of society, showing that vaccination is morally good). Overall, making the social contract explicit may help to increase vaccine uptake rates without relying on mandates, which seems to reflect the preferences of individuals (31) and would prevent countries from introducing selective mandates that could potentially decrease uptake for other voluntary vaccines (32).

However, there is also a potential downside to vaccination as a social contract. Individuals tend to accept social contracts if others do so as well (33). However, the media often reports that nonvaccination is becoming more and more common (e.g., ref. 34). The results of the present studies suggest that this narrative could negatively affect generosity and warmth, especially in vaccinated individuals. Both variables are related to cooperation and helping behavior (35, 36). As vaccination is a prosocial act itself (11), low levels of generosity and warmth can limit future willingness to be vaccinated. This means that overcommunicating the prevalence of nonvaccinated individuals could jeopardize high vaccine uptake, as the social contract requires trust in others’ compliance. We did not test this implication, but it is an important starting point for future research.

While the current results showed that people privilege those who obey the social contract irrespective of their group membership in real life, belonging to a specific group may often be confounded with a specific health status—migrants, for example, may have limited access to health care and thus be unwillingly forced to violate the social contract. It is also possible that other behaviors that help to control communicable diseases are seen as a social contract, such as wearing masks, hygiene measures, social distancing, etc. This may explain why in the current outbreak of COVID-19 discrimination and harassment of people of Asian descent can be observed (37), assuming that people of Asian descent violate the social contract of protecting others simply by being present. This real or perceived entanglement of social categories and health status can thus fuel existing stereotypes (38), leading to further marginalization and less positive behaviors from those who obey the contract. Thus, equitable access to health care and vaccinations is of utmost importance (39, 40) to allow everyone to fulfill the social contract and also to avoid further discrimination based on health status. Moreover, it is important to further explore the social contract hypothesis for other behaviors that prevent communicable diseases.

### Limitations.

Our research has some limitations. First, the data collection was conducted online, an environment in which participants are more prone to distraction from tasks and written instructions. However, previous research showed that the Amazon Mechanical Turk samples are superior to student and panel samples regarding their data quality and replicability of effects (41), as well as with regard to participants’ attentiveness (42).

Second, the experiment used a minimal group paradigm to allocate individuals to groups, which may imply reduced external validity of the results. A recent metaanalysis, however, showed no evidence for different in-group favoritism comparing natural versus minimal groups (18). Additionally, there was an intergroup bias in all experiments where group membership was manipulated, indicating that the group manipulation was successful. Future research could vary group types (natural vs. minimal) and examine actual previous vaccination behavior when researching changes of generosity in the vaccination context in order to further increase the external validity of the research.

Third, in this series of experiments, participants did not receive real vaccinations. However, the decision in favor or against vaccination (experiment 4: against or in favor of option A) yielded real monetary consequences experienced by the participants. Thus, it can be assumed that these incentivized decisions match real-world decisions better than purely hypothetical behavioral intentions (11, 43). Using real incentives has another benefit: experiments 2 and 3 examined changes of generosity in the context of migration. Migration is a sensitive topic (44), and, thus, it could be argued that socially desirable responses are especially prevalent in these experiments. Incentivized behavioral responses are less prone to socially desirable responses and “cheap talk” (26) and should therefore be less influenced by the migration framing as behavioral intentions.

### Conclusion.

The present research supports the notion that vaccination is a social contract wherein getting vaccinated is the morally right behavior. Future interventions should harness this finding to increase vaccine uptake. Moreover, the results also underline that equitable access to health systems and service delivery is of utmost importance to avoid further marginalization of already marginalized groups.

## Materials and Methods

### Ethics Statement.

The studies included human subjects and were conducted in accordance with the guidelines of the German Psychological Association. The studies did not involve deception. All participants gave written informed consent to use and share their data for scientific purposes without disclosure of their identity. The participants were free to quit the study at any time without any consequences. The experiment was conducted at a German university where institutional review boards or committees are not mandatory. The series of experiments was confirmed negligible risk research and therefore exempt of ethical approval, as declared by the University of Erfurt review board.

### Experiments 1 through 3.

#### Participants and design.

All experiments used a 2 × 2 × 2 quasiexperimental mixed design with participant’s vaccination decision (nonvaccination vs. vaccination, quasiexperimental, between), others’ vaccination decisions (nonvaccination vs. vaccination, within), and others’ group membership (in-group vs. out-group, within) as factors. Experiment 1 additionally varied the outcome interdependence of both groups (independent vs. interdependent, between), and participants were randomly allocated to the two conditions. Amazon Mechanical Turk users from the United States and Great Britain with an approval rate of 97% or higher were eligible for participation. The experiments were programmed with EFS Survey and were preregistered (experiment 1: https://aspredicted.org/5dp4n.pdf, experiment 2: https://aspredicted.org/9cw6u.pdf, experiment 3: https://aspredicted.org/rn3bs.pdf).

For experiment 1, an a priori power analysis, with an assumed small-to-medium effect size of the four-way interaction with *f* = 0.175 and a statistical test power of 1-β = 0.90 in a mixed effects ANOVA, revealed a target sample size of *n* = 168 participants. Due to the exclusion criteria (see below) and equally allocating participants to the conditions, we aimed to recruit 190 participants (*n* = 95 in each condition of the experimental between factor). The explorative analysis in experiment 1 uncovered an accidental and unexpected confound of vaccination attitude and the interdependence factor (there were significantly more participants with a negative attitude in the interdependence condition). We decided to proceed with recruitment until this confound was resolved, and this resulted in a total sample size of *n* = 242 individuals. For the mixed effects ANOVA in experiments 2 and 3, an a priori power analysis with *f* = 0.182 (derived from experiment 1, three-way interactions) and 1-β = 0.95 suggested a required total sample size of *n* = 132 participants. Moreover, experiment 1 showed that the majority of the participants decided to get vaccinated. As one’s own vaccination status was a quasiexperimental factor, we aimed to reach at least *n* = 66 participants also in the nonvaccination condition. Considering the potential exclusions, we recruited *n* = 146 (i.e., 78 participants in each of the conditions of the quasiexperimental factor) and continued to recruit participants until this number was reached.

The following exclusion criteria were preregistered for the experiments: incomplete participation, inappropriate participation time (upper and lower 5% quantile), and incorrect answers to attention check questions (see online materials for exact wording: https://osf.io/bn56v/).

Overall, *n* = 1,275 participants completed the three experiments. According to the preregistered exclusion criteria, we excluded from further analyses: *n* = 26 in experiment 1, *n* = 107 in experiment 2, and *n* = 110 in experiment 3. Thus, the final sample consisted of *n* = 1,032 (experiment 1: *n* = 216; experiment 2: *n* = 372; experiment 3: *n* = 444). For descriptive data on demographics and psychological characteristics, see *SI Appendix*, Table S8 in the supplement.

#### The one-shot I-Vax game.

In the one-shot I-Vax game, the participants were endowed with 100 fitness points (converting to $0.20) representing their health status (11). The participants were informed that 125 respondents were taking part in the study and that each of them would be allocated either to group A (group size = 95) or group B (group size = 30). All participants were then assigned to group A. Members of group B (the out-group) were not part of the main data collection but were collected after the main study using Mechanical Turk. All participants were paid based on their own vaccination decision and a randomly selected other individual. This procedure was done to ensure decision-compatible payment of the participants.

In experiment 1, depending on the interdependence condition, the participants learned that either both groups’ vaccination decisions or only group A’s vaccination decision affected the respondent’s payoff. The participants were confronted with a fictitious disease and had the opportunity to get vaccinated against this disease. A decision in favor of vaccination yielded fixed costs of 10 fitness points, resembling costs such as waiting time. Vaccine-adverse events occurred with a probability of 45%, leading to a loss of 15 points when they occurred. The expected costs of vaccination were thus 16.75 fitness points. A decision against vaccination yielded no fixed costs. However, nonvaccinated individuals were at risk for contracting the disease. The probability of infection was calculated based on the variable vaccination rate in the population and the fixed contagiousness of the disease (basic reproduction number R_0_ = 3). When infected, participants lost 50 fitness points. All this was known to the participants (see instruction materials on the Open Science Framework: https://osf.io/bn56v/).

In summary, the parametrization of the game implies a Nash equilibrium (45) at a vaccination rate of 50%, meaning that no participant has an incentive to change his or her strategy unilaterally at this vaccination rate (for visualization see ref. 12). Below a vaccination rate of 50%, vaccination is the dominant strategy; above a 50% vaccination rate, nonvaccination is the dominant strategy. However, collective welfare is maximized when 67% of the population decides in favor of vaccination. This yields the social optimum because, at this percentage, the infection probability reaches zero and the disease is eliminated. Thus, between the range of a 50% and 67% vaccination rate, the game constitutes a social dilemma in which individual interests are in conflict with collective interests. The participants’ vaccination decision in the one-shot I-Vax game (coded as 0 = nonvaccination, 1 = vaccination) served as a quasiexperimental factor in the analysis.

#### Experimental factors.

Before assessing conditional generosity, participants were reminded of their group membership by a figure presented to them. They were informed about the other’s group membership (coded 0 = in-group, 1 = out-group) and the other’s vaccination decision (0 = nonvaccination, 1 = vaccination) when the dependent variable was assessed. This procedure is described below.

In experiment 1, the participants were additionally informed whether the other group will influence their payment or not. In the independence condition (coded as 0), members of group A and group B constitute two separate, independent populations: “your payment will be affected by your decision, and the decisions of the members of your group A. Group B is irrelevant for your additional payment.” In contrast, in the interdependence condition (coded as 1), members of group A and group B were outcome-interdependent: “your payment will be affected by your decision, the decisions of the members of your group A, and the decisions of the members of the other group B.” The analysis of experiment 1 (*SI Appendix*, Fig. S2 and Table S3) also confirmed the social contract hypothesis. Furthermore, the analysis showed that vaccinated individuals in the independence condition showed lower levels of generosity, but to a lesser degree than individuals in the interdependence condition. In experiments 2 and 3, the groups were always interdependent.

#### Migration framing.

The framing of the decision situation varied across the experiments. In experiments 1 and 2, neutral group names A and B (in-group and out-group, respectively) were used. In experiment 3, group A was framed as the “host population,” and group B was framed as the “migrating group.” In experiments 2 and 3, participants received an animated figure (see online materials: https://osf.io/bn56v/), indicating that the out-group was migrating into the in-group, visualizing the outcome interdependence between the groups.

#### Dependent variable.

Generosity was assessed via the social value orientation slider measure (25), consisting of a sequence of six decision tasks. Each respondent allocated points (100 points = US$0.20) between himself or herself and another participant (receiver). All decisions were made from the perspective of the sender (strategy method, ref. 46). At the end of the experiment, one of the six allocation decisions in each block became payoff relevant for the sender and the matched recipients. The role was chosen randomly. The responses were transformed into a single index of a participant’s social value orientation, with higher values indicating more generosity.

The main dependent variable was change in generosity. Changes in generosity were assessed by measuring participants’ generosity twice. The baseline measurement at the beginning of the experiment was the unconditional social value orientation, where participants had no information about the receiver. After the one-shot I-Vax game, conditional generosity was measured such that participants received additional information about the receiver’s vaccination decision and group membership. All four combinations were assessed within subjects, i.e., vaccinated in-group member, nonvaccinated in-group member, vaccinated out-group member, and nonvaccinated out-group member. For the analyses, the baseline measurement was subtracted from the conditional measurements.

#### Perceived warmth.

In experiments 2 and 3, perceived warmth regarding the in-group and the out-group, and individuals of the four specific subgroups was measured. This was done using the feeling thermometer (47), by which participants indicated their perceived warmth on a scale ranging from 0 °F (very cold) to 100 °F (very warm).

#### Additional variables.

In all experiments, the following variables were assessed for explorative purposes, but were not part of the analysis: attitude toward vaccination (three items, e.g., “It is a good idea to get vaccinated,” adapted from ref. 48 on a seven-point Likert-type scale ranging from 1 = fully disagree to 7 = fully agree); identification with the in-group (four items, e.g., “I am glad to be part of group A,” adapted from ref. 49 on a seven-point Likert-type scale ranging from 1 = not at all to 7 = very much). In addition, beliefs about vaccine uptake in both groups were assessed in an open, numeric answer format (value between 0 and 100; e.g., “I think that [insert number]% of the other group B will choose to get vaccinated.”).

#### Attention checks and comprehension questions.

In experiment 1, the attention check question was presented after the first assessment of generosity (50). Incorrect answers led to immediate exclusion from the experiment (*n* = 140 participants were screened out). Experiments 2 and 3 included two attention check questions based on ref. 51 (see instructions on the Open Science Framework for exact wording: https://osf.io/bn56v/). In contrast to experiment 1, in experiments 2 and 3, incorrect answers led to preregistered exclusion from the analysis but not from participating and being paid (experiment 2: *n* = 70, experiment 3: *n* = 59).

After the participants received the instructions of the one-shot I-Vax game, they were asked to answer comprehension questions regarding the game. If, and only if, these responses were correct, could participants proceed with the study. However, wrong answers could be corrected, and the participants had the opportunity to download a pdf file containing the instructions of the one-shot I-Vax game.

#### Procedure.

Instructions for all experiments are available via the Open Science Framework (https://osf.io/bn56v/).

#### Payment.

The participants received a fixed ($2) payment and a bonus payment via Mechanical Turk. The bonus payment was: $0.77 (*SD* = 0.09) in experiment 1, $0.90 (*SD* = 0.11) in experiment 2, and $0.91 (*SD* = 0.10) in experiment 3. Bonus payments varied and were contingent on the decisions made in the game and the answers in the five blocks that assessed the social value orientation (one of the six allocation decisions in each block was payoff relevant).

The members of the out-group (group B) also participated in the social value orientation measure and played the I-Vax game. They received a fixed remuneration of $0.67 and a decision-contingent bonus payment, based on the outcomes of the social value orientation measure and the one-shot I-Vax game. Their average bonus payment was: $0.34 (*SD* = 0.04) in experiment 1, $0.31 (*SD* = 0.05) in experiment 2, and $0.33 (*SD* = 0.05) in experiment 3.

### Experiment 4.

#### Participants and design.

The experiment implemented a 2 × 2 × 2 × 2 quasiexperimental mixed design with participant’s decision (nonvaccination [not option A] vs. vaccination [option A], quasiexperimental, between), others’ decision (nonvaccination [not option A] vs. vaccination [option A], within), framing (neutral vs. vaccination, between), and mutual dependence (absent vs. present, between) as factors. Participants were randomly allocated to the four conditions of the between-subjects factors. The same Amazon Mechanical Turk inclusion criteria as in experiments 1 through 3 were applied. EFS survey was used for programming.

The a priori power analysis (https://aspredicted.org/m4yj7.pdf; effect size of the four-way interaction: *f* = 0.175, statistical test power of 1-β = 0.95 in a mixed effects ANOVA, given α = 0.05) revealed a target sample size of *n* = 368 participants. As in the other experiments, due to the exclusion criteria and a desired equal distribution of participants to the conditions, we preregistered recruitment of 448 participants (at least *n* = 56 participants in each cell of the experimental design). As we also needed 56 participants who decided against vaccination (or option A) and the inclination to vaccinate (choose option A) was relatively high, 1,558 participants had to complete the experiment. According to the preregistered exclusion criteria we removed *n* = 346 from further analyses. Thus, the final sample consisted of *n* = 1,212 (for descriptive data see *SI Appendix*, Table S8).

#### Experimental factors.

Framing and mutual dependence were varied in the one-shot I-vax game as follows.

##### Framing.

The material for the framing conditions was based on materials from a previous experiment on framing effects in the I-Vax game (11). The experimental setup varied the labeling of the decision task either as a decision in favor or against vaccination (vaccination framing; coded as 1) or in favor or against option A (neutral framing; coded as 0). This means, in the framing condition terms such as infection, vaccination, and side effects were used. The neutral framing condition implemented neutrally framed equivalents (see material at OSF for exact wording: https://osf.io/bn56v/).

##### Mutual dependence.

For readability purposes, the variation of the material regarding the experimental factor mutual dependence is presented from the perspective of the vaccination framing only. Before introducing the decision task, participants were informed that 112 participants perform the task as well and the decisions of all participants in this task are independent from each other (mutual dependence absent; coded as 0) or that they affect one another (mutual dependence present; coded as 1). In the mutual dependence condition, participants then played the one-shot I-Vax game with the exact same parametrization as in experiments 1, 2, and 3.

In the condition without mutual dependence, the decision task differed from the mutual dependence condition only with respect to the nonvaccination option. Deciding against vaccination in the mutual dependence conditions implies that the risk of infection is uncertain and falls into a certain range, depending on the personal beliefs about the choices of the other players. To make the conditions equivalent, we induced the same uncertainty regarding the probability of infection also in the condition where decisions were not mutually dependent. Participants were informed that the infection probability varies for unknown reasons and ranges between 0% and 67%. The range was calculated based on the beliefs about vaccine uptake in the previous experiments. As in the mutual dependence condition, infection resulted in a loss of 50 fitness points.

#### Dependent variable.

The same procedure as in the previous experiments was applied. At the beginning of the experiment, unconditional generosity was measured. After the focal decision task, participants received additional information about the other’s decision and were reminded about the absence vs. presence of mutual dependence between participants. Then generosity was measured again, this time conditional on the other’s characteristics. For the analyses, the baseline measurement was again subtracted from the conditional measurements.

#### Additional variables.

Perception of vaccination as a moral obligation was measured with four self-developed items (e.g., “There is a great deal of agreement that vaccination is the morally good thing to do.”) on a seven-point Likert-type scale ranging from 1 = fully disagree to 7 = fully agree. Internal consistency of the scale was excellent (α = 0.93).

#### Procedure.

The full instructions are available via the Open Science Framework (https://osf.io/bn56v/).

#### Payment.

The participants received a fixed ($2) payment and a bonus payment via Mechanical Turk. The bonus payment was: $0.61 (SD = 0.08) and varied contingent on the decisions made in the decision task and the answers in the three blocks that assessed social value orientation (one of the six allocation decisions in each block was payoff relevant).

### Data Analysis.

The R-environment (52) and the R-packages *lme4* (53) and *metafor* (54) were utilized for the metaanalysis. Although the hypotheses proposed were directional, conservative two-sided tests were used. An alpha level of 0.05 for all analyses was applied.

The metaanalysis was based on mixed effects regressions with the predictors being the participant’s vaccination decision, other’s vaccination decision, group membership, and their interactions on changes in generosity for each experiment (*SI Appendix*, Table S1). The estimated effects of the mixed effects regressions and their SEs were standardized. For each effect, 95% confidence intervals were computed. To account for the differences in the materials between the experiments, a random effects model for the metaanalysis was used. Across the effects, the Q statistics were not significant, indicating sufficient homogeneity. With regard to the three-way interaction, however, the proportion of observed variance across the studies, reflected by I^2^, was moderate. Omission of experiment 2 led to the strongest reduction of I^2^ but to qualitatively identical results. Thus, experiment 2 was not removed from the analysis.

### Data Availability.

The materials, data, and syntax of all four experiments are available online from the Open Science Framework (https://osf.io/bn56v/).

## Supplementary Material

Supplementary File
