# Supplementary material for: Vaccination as a social contract
Source: Proc Natl Acad Sci U S A. 2020 Jun 15;117(26):14890–9. doi: 10.1073/pnas.1919666117 (PMC7334515; doi:10.1073/pnas.1919666117)
Supplement: Supplementary File [file pnas.1919666117.sapp.pdf]

PNAS
www.pnas.org

Supplementary Information for

Vaccination as a social contract

Lars Korn, Robert Böhm, Nicolas W. Meier, and Cornelia Betsch

Lars Korn
Email: Lars.Korn@uni-erfurt.de

This PDF file includes:

Supplementary text
Figures S1 to S5
Tables S1 to S8

Results of the individual experiments

Experiment 1. The first experiment examined changes in generosity towards others based on the participant's vaccination behavior, vaccination behavior of the other, group membership, and a situation where two groups were either outcome independent from each other or outcome interdependent. Table S3 shows the results from the pre-registered repeated-measures ANOVA with the changes in generosity as a dependent variable. Figures 1 and S1 visualize the results across all experiments (Figure 1 in the main document provides the hypothesis tests and manipulation check; Figure S1 visualizes the remaining effects; and Figure S2 shows the results of Experiment 1 only). Figure S2 shows that, in accordance with the social contract hypothesis, vaccinated individuals especially reduced their generosity toward others who did not get vaccinated in the I-Vax game (M_{no_vacc} = -8.04, SD_{no_vacc} = 14.52) as compared to others who did get vaccinated (M_{vacc} = 0.97, SD_{vacc} = 9.75). Non-vaccinated participants, in contrast, did not differentiate between vaccinated and non-vaccinated others (M_{vacc} = -2.20, SD_{vacc} = 11.80, M_{novacc} = -2.95, SD_{novacc} = 11.17; interaction B*D in Table S3).

Moreover, the results revealed an intergroup bias. Individuals showed more generosity towards in-group members (*M* = -2.78, *SD* = 12.25) compared to out-group members (*M* = -3.83, *SD* = 13.26). We found, however, that this effect was moderated by interdependence, participant's vaccination decision, and other's vaccination decision.

The analysis further revealed a 4-way interaction (A*B*C*D in Table S3) on changes in generosity. Individuals take other's previous vaccination behavior into account, and do so to a stronger degree when the other's group matters for the own outcome. Figure S2 shows that non-vaccinated participants (grey diamonds) do not condition their generosity toward others on the other's vaccination decision, the other's group membership, or the interdependence condition. Moreover, all 95% CIs cross the zero-line, indicating no change from baseline to the conditional assessments of generosity. In contrast, vaccinated participants (black diamonds) show an increase (decrease) of generosity toward vaccinated (non-vaccinated) others.

In order to explore the pattern among vaccinated participants, simple main effects for other's vaccination decision were calculated separately for each of the four quadrants in Figure S2 (combinations from the other's group membership and interdependence). When outcomes are independent (upper two quadrants), generosity change towards in-group members (left, M_{vacc} = 1.88, SD_{vacc} = 10.27, M_{novacc} = -5.43, SD_{novacc} = 13.09; $F[1,83] = 22.29$, $p < .001$, $\eta^2_g = .09$) is quite similar to prosociality towards out-group members (right, M_{vacc} = 0.82, SD_{vacc} = 10.13, M_{novacc} = -5.89, SD_{novacc} = 14.22; $F[1,83] = 25.41$, $p < .001$, $\eta^2_g = .07$). When both groups are interdependent (lower two quadrants), generosity change is less pronounced with members of the in-group (left, M_{vacc} = 0.84, SD_{vacc} = 9.58, M_{novacc} = -9.12, SD_{novacc} = 14.46; $F[1,79] = 33.51$, $p < .001$, $\eta^2_g = .14$) as compared to the out-group (right, M_{vacc} = 0.31, SD_{vacc} = 9.03, M_{novacc} = -11.98, SD_{novacc} = 15.55; $F[1,79] = 43.71$, $p < .001$, $\eta^2_g = .19$).

These results indicate that an increase (decrease) of generosity toward vaccinated (non-vaccinated) others occurs especially toward out-group members when the groups are outcome interdependent. Nevertheless, this effect also occurred in the independence condition, which indicates that vaccination as a social contract also applies to individuals from an independent out-group.

Experiment 2. The second experiment aimed at replicating the results and visualized the interdependence of the groups by a gif that showed how the out-group B migrated into the ingroup A. Figure S3 visualizes the results; Table S4 shows the results from the pre-registered repeated-measures ANOVA with changes in generosity as the dependent variable. Similar to Experiment 1, there was evidence for the social contract hypothesis. Vaccinated individuals showed less generosity toward others who did not get vaccinated (M_{no_vacc} = -12.01, SD_{no_vacc} = 19.22) compared to others who got vaccinated (M_{vacc} = 0.92, SD_{vacc} = 10.15). Non-vaccinated participants, in contrast, did not differentiate between vaccinated and

non-vaccinated others (M_{vacc} = -0.44, SD_{vacc} = 13.97, M_{novacc} = -2.24, SD_{novacc} = 14.15; see Table S6, interaction A*C).
Again, the results revealed an intergroup bias. Individuals showed more generosity towards in-group members (*M* = -3.79, *SD* = 16.37) compared to out-group members (*M* = -5.52, *SD* = 16.07). This effect was qualified by an interaction with participant's vaccination decision (A*C) and other's vaccination decision (B*C).

Moreover, the analysis revealed a 3-way interaction (A*B*C in Table S4). An increase (decrease) of generosity toward vaccinated (non-vaccinated) others among vaccinated participants was moderated by group membership. This effect was more pronounced toward in-group members (left panel) than toward out-group members (right panel). Moreover, the 95% CI (see Figure S3) regarding generosity change from vaccinated participants toward vaccinated in-group members did not cross the zero-line, indicating a positive change from baseline to the conditional assessment of generosity. This pattern indicated rewarding behavior.

Experiment 3. The third experiment aimed to replicate the finding from Experiment 2 and used an explicit migration framing. Similar to Experiments 1 and 2, the data supported the social contract hypothesis (see Table S5 and Figure S4). The results showed that vaccinated individuals reduced their generosity toward others who did not get vaccinated in the I-Vax game (M_{no_vacc} = -11.17, SD_{no_vacc} = 16.69) compared to others who got vaccinated (M_{vacc} = 1.23, SD_{vacc} = 10.10). Non-vaccinated participants, in contrast, did not differentiate between vaccinated and non-vaccinated others (M_{vacc} = -0.92, SD_{vacc} = 9.28, M_{novacc} = -3.53, SD_{novacc} = 9.73; see Table S5, interaction A*C).

Again, the results revealed an intergroup bias. Individuals showed more generosity toward in-group members (*M* = -3.83, *SD* = 13.91) compared to out-group members (*M* = -5.06, *SD* = 14.59). In contrast to Experiment 2, the increase (decrease) of generosity toward vaccinated (non-vaccinated) others among vaccinated participants was not moderated by group membership.

Experiment 4. The fourth experiment investigated whether mutual dependency (absent vs. present) and framing (neutral vs. vaccination) moderate the social contract effect (interaction between participant's and other's decision). Figure S5 visualizes the results; Table S6 shows the results from the pre-registered repeated-measures ANOVA with changes in generosity as the dependent variable. The results showed that individuals who opted in favor of vaccination (or option A) reduced their generosity toward others who decided against vaccination (or option A; $M_{against}$ = -2.78, $SD_{against}$ = 11.67) in the decision task compared to others who opted in favor of vaccination (or option A; M_{in_favor} = 0.10, SD_{in_favor} = 8.95). In contrast, participants who decided against vaccination (or option A) themselves, did not differentiate between others depending on their decision (M_{in_favor} = -1.73, SD_{in_favor} = 10.59, $M_{against}$ = -1.50, $SD_{against}$ = 10.32; see Table S6, interaction A*B). Contrary to the pre-registered hypotheses, this interaction effect was neither moderated by framing nor by mutual dependence (see Table S6, interaction A*B*C, A*B*D respectively).

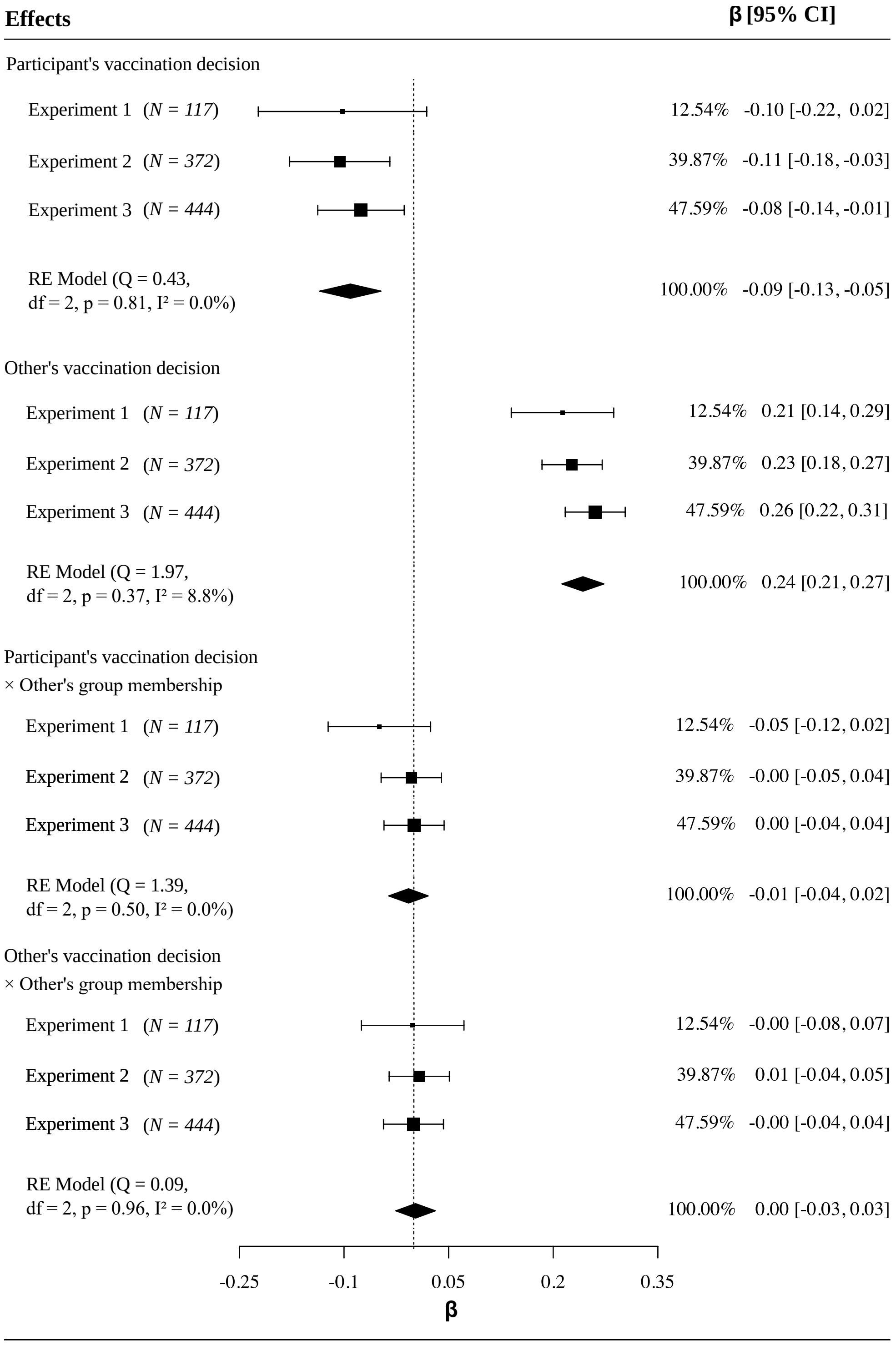

Fig. S1. Forest plot of the effects of participant's vaccination decision, other's vaccination decision, interaction between participant's vaccination decision and other's group membership, and interaction between other's vaccination decision and other's group membership on generosity change. Effects displaying betas, calculated from mixed effects regressions, and overall effects using a random effects model for meta-analysis. CIs refer to 95% confidence intervals. Q and I^2 were used for a heterogeneity assessment among studies.

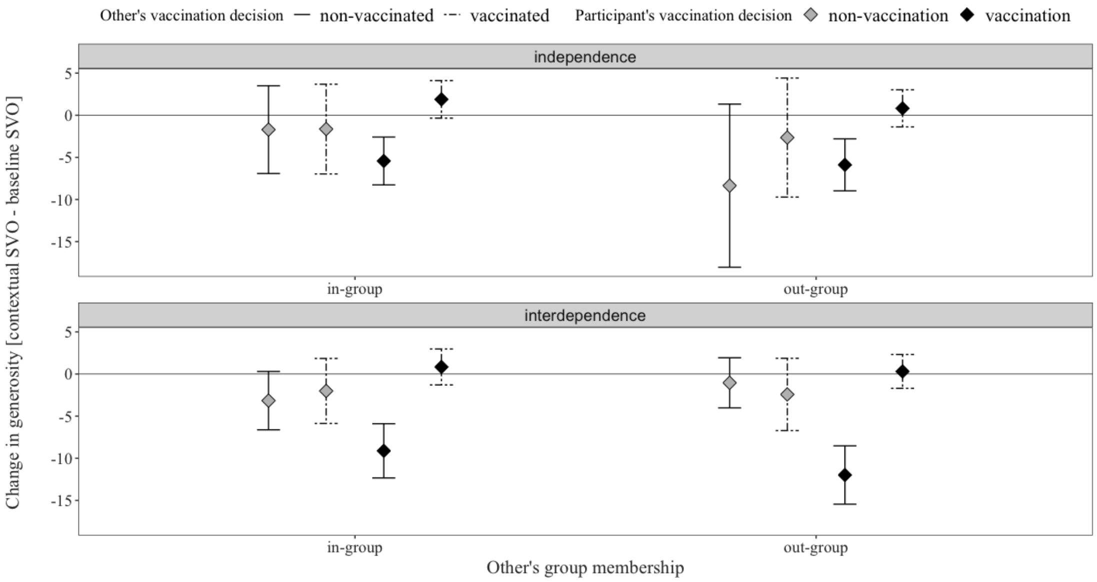

Fig. S2. Changes in generosity as a function of interdependence, other's group membership, other's vaccination decision, and participant's vaccination decision in Experiment 1. The factor of interdependence was used to split the errorbar plot. Diamonds show the mean change of generosity; errorbars represent 95% CIs. *N* = 216. Note: Non-vaccinated participants (grey errorbars) do not change their generosity based on the other's vaccination behavior, group membership, and interdependence. Vaccinated participants (black errorbars) condition their generosity on the other's vaccination behavior. Vaccinated participants reduce their generosity toward non-vaccinated others (solid errorbars) compared to vaccinated others (dashed errorbars). This effect is more pronounced toward out-group members (right side of the plot) when both groups were outcome interdependent.

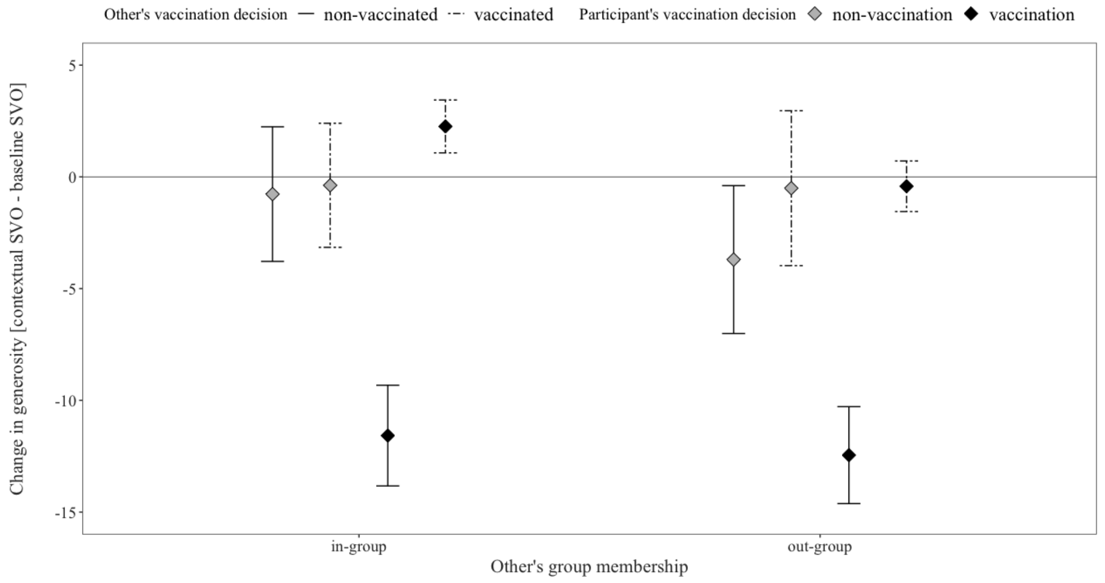

Fig. S3. Changes in generosity as a function of interdependence, other's group membership, other's vaccination decision, and participant's vaccination decision in Experiment 2. Diamonds show the mean change of generosity; errorbars represent 95% CIs. $N = 372$. Note: Non-vaccinated participants (grey errorbars) do not adapt their generosity based on the other's vaccination behavior and group membership. Vaccinated participants (black errorbars) condition their generosity on the other's vaccination behavior.

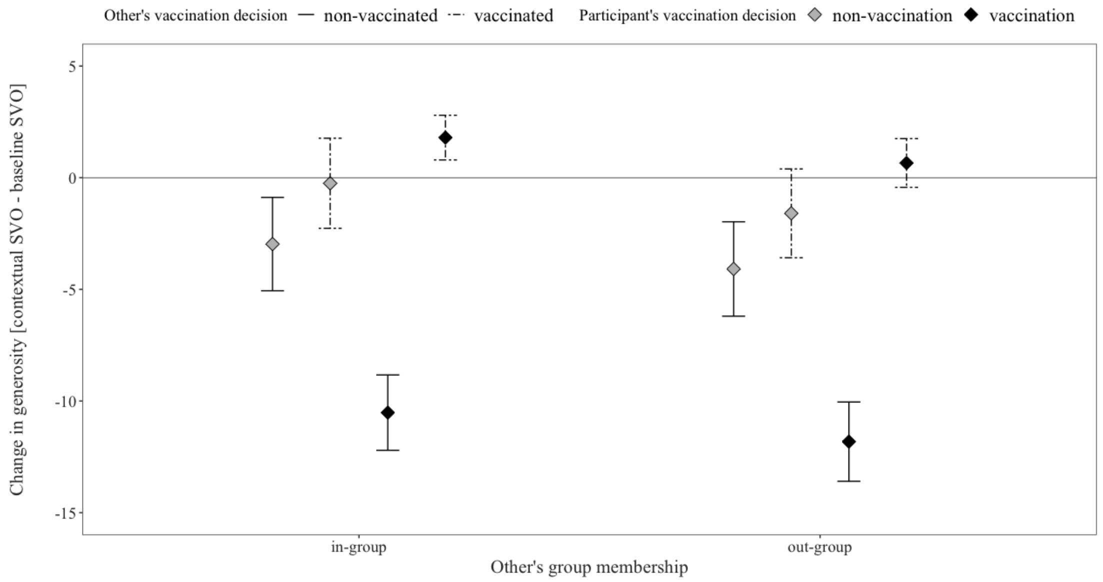

Fig. S4. Changes in generosity as a function of interdependence, other's group membership, other's vaccination decision, and participant's vaccination decision in Experiment 3. Diamonds show the mean change of generosity; errorbars represent 95% CIs. N = 444. Note: Generosity change was more pronounced among vaccinated participants (black errorbars). They condition their generosity on the other's vaccination behavior, but not on the other's group membership.

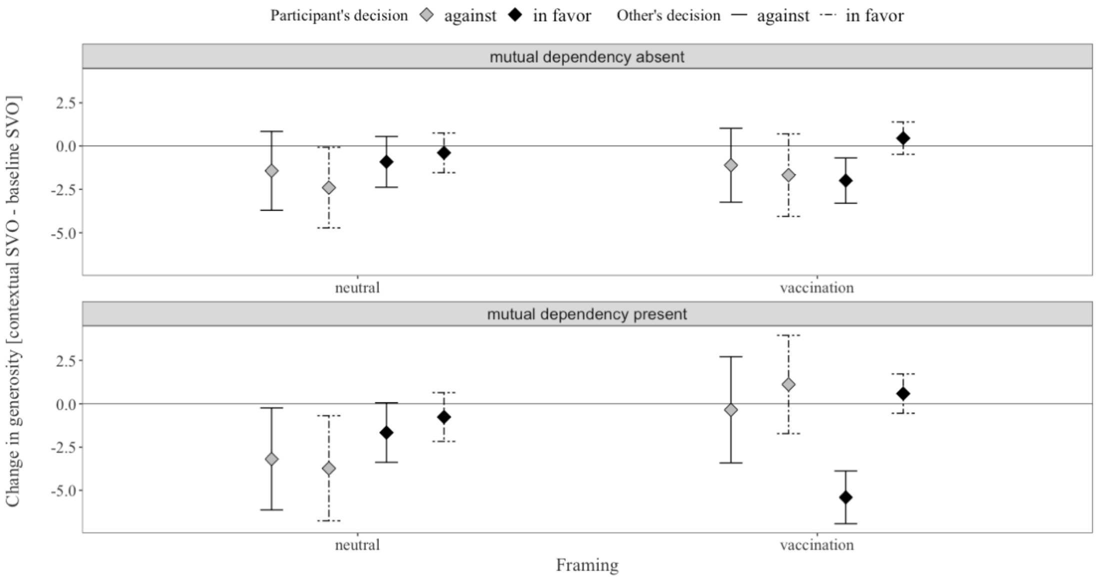

Fig. S5. Changes in generosity as a function of participant's vaccination decision, other's vaccination decision, framing, and mutual dependence in Experiment 4. Errorbars represent 95% CIs. *N* = 1,212. Note: Generosity change was more pronounced among vaccinated participants (black errorbars), irrespective of framing and mutual dependency (Table S6).

Table S1. Mixed effects models predicting change in generosity as a function of participant's vaccination decision, other's vaccination decision, other's group-membership, and their interactions, separate for each experiment.

	Experiment 1			Experiment 2			Experiment 3		
Predictors	*B*	*SE*	*p*	*B*	*SE*	*p*	*B*	*SE*	*p*
Intercept	-3.57	0.86	<.001	-3.44	0.73	<.001	-3.60	0.57	<.001
Participant's vaccination decision (A)	-2.83	1.71	.101	-4.21	1.46	.004	-2.75	1.15	.017
Other's vaccination decision (B)	5.50	0.97	<.001	7.36	0.72	<.001	7.50	0.63	<.001
Other's group membership (C)	-0.42	0.97	.662	-1.65	0.72	.021	-1.22	0.63	.051
A*B	11.24	1.94	<.001	11.14	1.43	<.001	9.79	1.25	<.001
A*C	-2.55	1.94	.189	-0.25	1.43	.863	0.01	1.25	.993
B*C	-0.09	1.94	.962	0.50	1.43	.727	-0.03	1.25	.978
A*B*C	4.87	3.87	.210	-4.60	2.86	.108	0.39	2.50	.876
Observations / N	468 / 117			1,488 / 372			1,776 / 444		
Marginal R^2 / Conditional R^2	.141 / .439			.140 / .519			.161 / .472		

Note. *Mixed effects model (prediction of generosity change): Participants treated as random effect. Effect coding was used for all predictors. Participant's vaccination decision: -.5 = non-vaccination, +.5 = vaccination. Other's vaccination decision: -.5 = non-vaccination, +.5 vaccination. Group membership: -.5 = in-group, +.5 = out-group. Marginal R^2 refers to the proportion of variance explained by the fixed factors. Conditional R^2 refers to the proportion of variance explained by the fixed factors and the random factor.*

Table S2. Mixed effects model predicting warmth toward others as a function of the factors of participant's vaccination decision, other's vaccination decision, other's group membership, and their interactions in Experiments 2 and 3.

	**Warmth**		
Predictors	β	*SE*	*p*
Participant's vaccination decision (A)	-0.05	0.02	<.001
Other's vaccination decision (B)	0.48	0.02	<.001
Other's group membership (C)	-0.08	0.02	<.001
Experiment (D)	-0.01	0.02	.438
A*B	0.22	0.02	<.001
A*C	0.02	0.02	.326
B*C	0.01	0.02	.751
A*B*C	-0.03	0.02	.078
Observations / N		3,264 / 816	
Marginal R^2 / Conditional R^2		.418 / .482	

Note. Mixed effects model (warmth): Participants treated as random effect. Effect coding was used for all predictors. Participant's vaccination decision: -.5 = non-vaccination, +.5 vaccination. Other's vaccination decision: -.5 = non-vaccination, +.5 vaccination. Group membership: -.5 = in-group, +.5 out-group. Experiment: -.5 = Experiment 2, +.5 = Experiment 3. Marginal R^2 refers to the proportion of variance explained by the fixed factors. Conditional R^2 refers to the proportion of variance explained by the fixed factors and the random factor.

Table S3. Results from Univariate Type III Repeated-Measures ANOVA with assumed sphericity in Experiment 1: Changes in generosity as a function of the factors of participant's vaccination decision, other's vaccination decision, other's group membership, and interdependence.

Predictors	*df*	*MSE*	*F*	*p*	η^2_g
Participant's vaccination decision (A)	1, 212	331.64	0.20	.657	<.001
Other's vaccination decision (B)	1, 212	174.66	21.18	<.001	.030
Other's group membership (C)	1, 212	44.00	5.69	.018	.002
Interdependence (D)	1, 212	331.64	0.20	.654	<.001
A*B	1, 212	174.66	11.43	.001	.020
A*C	1, 212	44.00	0.05	.821	<.001
A*D	1, 212	331.64	1.86	.174	.005
B*C	1, 212	35.44	1.40	.238	<.001
B*D	1, 212	174.66	0.06	.807	<.001
C*D	1, 212	44.00	2.69	.102	.001
A*B*C	1, 212	35.44	0.11	.735	<.001
A*B*D	1, 212	174.66	2.46	.119	.003
A*C*D	1, 212	44.00	6.08	.015	.002
B*C*D	1, 212	35.44	1.62	.204	<.001
A*B*C*D	1, 212	35.44	7.38	.007	.002

Note. N = 216*. df* indicates numerator and denominator degrees of freedom. η^2_g indicates generalized eta-squared.

Table S4. Results from Univariate Type III Repeated-Measures ANOVA with assumed sphericity in Experiment 2: Changes in generosity as a function of the factors of participant's vaccination decision, other's vaccination decision, and other's group membership.

Predictors	*df*	*MSE*	*F*	*p*	η^2_g
Participant's vaccination decision (A)	1, 370	528.75	8.34	.004	.010
Other's vaccination decision (B)	1, 370	277.34	48.65	<.001	.040
Other's group membership (C)	1, 370	58.24	11.67	.001	.002
A*B	1, 370	277.34	27.83	<.001	.020
A*C	1, 370	58.24	0.07	.798	<.001
B*C	1, 370	46.33	0.34	.563	<.001
A*B*C	1, 370	46.33	7.12	.008	<.001

Note. *N* = 372. *df* indicates numerator and denominator degrees of freedom. η^2_g indicates generalized eta-squared.

Table S5. Results from Univariate Type III Repeated-Measures ANOVA with assumed sphericity in Experiment 3: Changes in generosity as a function of the factors of participant's vaccination decision, other's vaccination decision, and other's group membership.

Predictors	*df*	*MSE*	*F*	*p*	η^2_g
Participant's vaccination decision (A)	1, 442	361.66	5.73	.017	.007
Other's vaccination decision (B)	1, 442	241.37	64.09	<.001	.050
Other's group membership (C)	1, 442	45.53	9.05	.003	.001
A*B	1, 442	241.37	27.29	<.001	.020
A*C	1, 442	45.53	<0.01	.989	<.001
B*C	1, 442	36.37	<0.01	.963	<.001
A*B*C	1, 442	36.37	0.07	.789	<.001

Note. N = 444. *df* indicates numerator and denominator degrees of freedom. η^2_g indicates generalized eta-squared.

Table S6. Results from Univariate Type III Repeated-Measures ANOVA with assumed sphericity in Experiment 4: Changes in generosity as a function of the factors of participant's vaccination decision, other's vaccination decision, framing, and mutual dependency, and their interactions.

Predictors	*df*	*MSE*	*F*	*p*	η^2_g
Participant's vaccination decision (A)	1, 1,204	157.06	0.28	.594	<.001
Other's vaccination decision (B)	1, 1,204	62.73	8.46	.004	.002
Framing (C)	1, 1,204	157.06	1.47	.226	<.001
Mutual dependency (D)	1, 1,204	157.06	0.61	.436	<.001
A*B	1, 1,204	62.73	10.85	.001	.003
A*C	1, 1,204	157.06	5.10	.024	.003
A*D	1, 1,204	157.06	0.94	.334	<.001
B*C	1, 1,204	62.73	8.79	.003	.002
B*D	1, 1,204	62.73	4.05	.044	.001
C*D	1, 1,204	157.06	0.79	.373	<.001
A*B*C	1, 1,204	62.73	2.11	.146	<.001
A*B*D	1, 1,204	62.73	0.21	.646	<.001
A*C*D	1, 1,204	157.06	3.06	.080	.002
B*C*D	1, 1,204	62.73	2.26	.133	<.001
A*B*C*D	1, 1,204	62.73	0.24	.624	<.001

Note. N = 1,212*. df* indicates numerator and denominator degrees of freedom. η^2_g indicates generalized eta-squared.

Table S7. Mixed effects model predicting change in generosity as a function of perception of vaccination as moral obligation, participant's vaccination decision, other's vaccination decision, framing, mutual dependence, and their interactions.

				Generosity change			
Predictors	β	*SE*	*p*	*Predictors*	β	*SE*	*p*
Moral obligation (A)	0.01	0.03	.764	A*B*D	-0.03	0.03	.274
Participant's decision (B)	0.00	0.03	.948	A*C*D	0.05	0.02	.006
Other's decision (C)	0.06	0.02	.002	B*C*D	0.01	0.02	.590
Framing (D)	0.04	0.03	.189	A*B*E	0.08	0.03	.003
Mutual dependence (E)	-0.03	0.03	.270	A*C*E	-0.01	0.02	.620
A*B	-0.04	0.03	.133	B*C*E	0.01	0.02	.603
A*C	0.07	0.02	<.001	A*D*E	0.05	0.03	.054
B*C	0.05	0.02	.019	B*D*E	-0.05	0.03	.126
A*D	-0.02	0.03	.424	C*D*E	0.03	0.02	.077
B*D	-0.06	0.03	.037	A*B*C*D	0.04	0.02	.034
C*D	0.06	0.02	.002	A*B*C*E	-0.03	0.02	.124
A*E	-0.03	0.03	.305	A*B*D*E	-0.03	0.03	.277
B*E	-0.02	0.03	.454	A*C*D*E	0.02	0.02	.304
C*E	0.04	0.02	.039	B*C*D*E	0.00	0.02	.835
D*E	0.02	0.03	.568	A*B*C*D*E	0.00	0.02	.876
A*B*C	0.00	0.02	.960				
Observations / N				2,424 / 1,212			
Marginal R^2 / Conditional R^2				.051 / .467			

Note. *Mixed effects model (prediction of generosity change): Participants treated as random effect. Effect coding was used for all factors. Participant's vaccination decision: -.5 = non-vaccination, +.5 = vaccination. Other's vaccination decision: -.5 = non-vaccination, +.5 vaccination. Framing: -.5 = neutral, +.5 = vaccination. Mutual dependence: -.5 = absent, +.5 = present. Predictor moral obligation was centered at the mean for the analysis. Marginal R^2 refers to the proportion of variance explained by the fixed factors. Conditional R^2 refers to the proportion of variance explained by the fixed factors and the random factor. The four-way interaction A*B*C*D is displayed in Figure 4 in the main text.*

Table S8. Demographics and psychological characteristics of the participants per experiment.

	Experiment 1		Experiment 2		Experiment 3		Experiment 4	
Variables	*M*	*SD*	*M*	*SD*	*M*	*SD*	*M*	*SD*
Age in years	35.70	10.02	34.56	9.58	36.25	10.89	36.46	11.00
Gender (% female)	42.1		42.7		43.0		42.1	
Participation time in minutes	12.74	4.13	14.82	5.58	16.61	5.75	12.08	4.73
Baseline SVO	22.38	13.67	20.85	14.45	23.73	14.30	21.66	13.80
Vaccination attitude	5.95	1.49	6.14	1.35	6.10	1.40		
Group identity	4.34	1.62	4.66	1.57	4.73	1.46		
Behavioral beliefs in-group	63.61	18.90	67.51	20.38	66.31	18.18		
Behavioral beliefs out-group	61.69	19.74	62.45	23.38	55.69	22.58		
Warmth toward in-group, pre			70.29	18.43	71.54	18.77		
Warmth toward in-group, post			69.54	17.80	71.48	17.49		
Warmth toward out-group, pre			59.29	17.93	58.39	18.04		
Warmth toward out-group, post			59.49	18.02	58.43	19.44		
Moral obligation							5.61	1.38

Note. Missing values regarding gender: Experiment 1 = 5, Experiment 2 = 8, Experiment 3 = 9.
